# Symptom vs context: lessons learned from a large-scale implementation of the Cultural Formulation Interview

**DOI:** 10.3389/fpsyt.2024.1410865

**Published:** 2024-09-04

**Authors:** Linda Silvius, Katrina V. Antezana J., Samrad Ghane

**Affiliations:** Parnassia Academy, Parnassia Psychiatric Institute, The Hague, Netherlands

**Keywords:** cultural formulation interview, cultural competences, disparity, inequity, mental health, cultural assessment

## Abstract

Mental health services in multicultural societies require culturally sensitive approaches to reduce health disparities. The Cultural Formulation Interview (CFI) is thought to enhance shared decision making and to facilitate culturally and contextually informed treatment. There is, however, little known regarding its implementability in large-scale psychiatric services. The current paper reports on (a) efforts to implement the CFI in a large organization for mental health services in the Netherlands, and (b) two studies that evaluated this implementation process and identified barriers to CFI adoption in clinical practice. Implementation of the CFI was facilitated by developing an online course, an advanced training of “team coaches”, (digital) resources, and integrating the CFI questions into the intake format. A preliminary evaluation revealed that the CFI was administered in only 13.2% of all intakes across the entire organization, with minimal utilization of training resources. *Study 1* aimed to investigate how the CFI was perceived by clinicians and stakeholders. A survey of 150 clinicians found a great lack of familiarity with the CFI and its purpose. While 67% reported partial CFI use, 50% saw no added value, and 61% deemed it relevant only for ethnic minorities. *Study 2* examined which patient and clinician variables were associated with adequate CFI use (i.e., correct documentation of the CFI information in initial intake reports). The sample consisted of 112 intakes of patients conducted by ten clinicians. Regression analysis showed a significant association between clinicians’ cultural competences and adequate CFI use, meaning that more culturally competent clinicians tended to generate better cultural assessments using the CFI. In addition, the CFI information was documented more adequately among patients who were unemployed at the time of assessment. In conclusion, implementation of the CFI requires a fundamental rethinking of the entire intake assessment, shifting it from a symptom-oriented approach towards a context- and person-centered one. Future trainings may benefit from embedding the CFI within a broader cultural competency training, rather than solely focusing on the CFI, which is currently the common practice.

## Introduction

It has been increasingly recognized that culture impacts every aspect of the clinical process: the expression of distress through specific symptoms (1), how these symptoms are understood and explained (2), coping and help seeking (3), and finally how therapists and patients position themselves in the healing relationship (4). Past studies have shown that clinicians (5) and approaches (5–7) that are more responsive to patients’ culture and context tend to generate better outcomes. However, the dominant diagnostic and treatment approaches within psychiatry remain oblivious to patients’ cultural and social contexts, and thus tend to decontextualize patients’ experiences. This is even more problematic for patients from minoritized and marginalized communities who, compared to other social groups, are more frequently exposed to social adversities, and hence are more vulnerable to develop mental disorders (8). As today’s societies grow in cultural diversity and social complexity, it has become increasingly important to incorporate culturally responsive approaches within mainstream psychiatric services.

One major step in this direction was the inclusion of the Outline for Cultural Formulation (OCF) in the fourth edition of the Diagnostic and Statistical Manual of Mental Disorders (DSM-IV) (9). The OCF consisted of a list of relevant topics to be systematically explored in contact with patients and their significant others with the aim of facilitating culturally informed diagnostic assessment and treatment planning (10). These topics include: (a) cultural identity of the individual, (b) cultural explanations of the individual’s illness, (c) cultural factors related to psychosocial environment and levels of functioning, and (d) cultural elements of the relationship between the individual and the clinician. Since its introduction, there have been multiple attempts to operationalize the OCF for use in clinical practice (11, 12). However, the resulting interviews lacked a uniform format, which made it difficult to infer general conclusions regarding their clinical utility (13). Furthermore, the time needed to administer lengthy interviews generated doubt as to the extent they are suitable for use in routine clinical practice where time constraints hamper comprehensive cultural assessment.

In an attempt to address the limitations of the previous OCF-based interviews, the Cross-Cultural Issues Subgroup of DSM-5 developed a new set of cultural assessment tools, called the Cultural Formulation Interview (CFI) (14). The CFI consists of three interviews: (a) a core version with 16 questions tapping into, and organized around the OCF topics, (b) an informant version that helps elicit the perspectives of the patient’s significant others, and (c) a number of supplementary interview modules that focus more elaborately on specific OCF topics (e.g., cultural identity, explanatory models, etc.) or specific patient populations (e.g., older adults, immigrants and refugees, etc.). The core interview of the CFI is designed as an integral part of routine clinical assessment with *all* patients across *all* settings, with the option of expanding it, using the supplementary modules, if the clinician requires more information on one or more OCF topics.

Previous research into the CFI has generated a number of important outcomes. First, there is evidence that the CFI is likely to enhance clinical communication. Two studies found that the CFI seemed to encourage clinicians to ask more open-ended questions, to better address patients’ concerns and to explain more about what patients can expect from their treatments (15, 16). In another study in an outpatient setting in Mexico, patients and clinicians reported that the CFI helped establish better rapport, empathy and trust between patients and providers (17). Despite their importance, however, it must be noted that the current evidence is solely based on qualitative analyses of limited data, and that larger studies that focus more specifically on the impact of the CFI on treatment process and outcome are still underway (18).

A major concern regarding the CFI is its implementability in everyday clinical practice. Early implementation studies during the DSM-5 field trials supported the feasibility, acceptability and clinical utility of the CFI according to both patients and clinicians (19). Interestingly, patients tended to evaluate the CFI more favorably than clinicians, although the latter group went on to perceive the interview as more valuable after gaining more experience. With regard to fidelity, i.e., the extent to which the CFI was administered as intended, the data showed that clinicians asked each question at least 87% of the times, with the majority of the questions asked 100% of the times (20).

Now a decade following the introduction of the CFI, there is still little known about its potential for large-scale implementation in mainstream psychiatric services. The CFI is designed to be a seamless and integrated part of clinical assessment, and at the same time to be “an agent of change”. That is, “to advance what is, in effect, a radical agenda: to change the way clinicians conduct a diagnostic interview so that the perspective of the patient becomes at least as important as the signs and symptoms of disease identified by the clinician” (21). The question is, however, to what extent psychiatry is receptive to such a radical approach. Furthermore, as psychiatric services attempt to adopt the CFI on a large scale, which barriers are encountered in its implementation.

The current paper aims to fill in this knowledge gap by presenting two studies that investigated (a) the implementation of the CFI within the largest provider of mental health services in the Netherlands, (b) how the CFI is perceived by different staff and stakeholders, and (c) factors that are associated with adequate use of the CFI during routine diagnostic assessment. In the first study, a mixed method design was incorporated to shed light on how the CFI and its implementation was perceived and understood by different actors in different organizational layers. Study 2 used historical cohort data to examine which patient and clinician variables were associated with greater CFI fidelity during initial assessments. Partly based on the findings in study 1, we hypothesized CFI fidelity to be greater among more culturally competent clinicians.

## Background and setting

Both studies were conducted at Parnassia Psychiatric Institute, the largest provider of mental health services in the Netherlands. At the time of the studies, the organization had approximately 12,000 employees, and provided support to more than 180,000 patients. Parnassia’s services include all psychiatric specialties at all levels of intensity and for all age groups, including adult ambulatory mental health services, assertive community treatment teams, clinical facilities, child and adolescent psychiatry, and geriatric mental health units. Each psychiatric specialty is managed by a separate board of directors and divided into subregions within the country. A central board of directors oversees the entire operations of the organization across all specialties. Since these services are mainly concentrated in large metropolitan areas with vastly culturally diverse populations, some 30% of all patients are known to have an immigration background.

Between 2011 and 2013 the organization participated in the international CFI field trials, as one of three centers in the Netherlands which provided data on the feasibility, acceptability and clinical utility of the instrument. In that very same period, the organization set out to transition from a symptom-oriented approach to a person-centered and recovery oriented one. This involved a shift away from a (DSM) classification based diagnosis and treatment planning towards a focus on patients’ personal needs, subjective experiences, perspectives, strengths and support systems. The CFI was deemed a useful instrument to facilitate this process. Thus, in 2017 the CFI became an integral part of every intake assessment regardless of the clinical setting or the cultural background of the patient.

To facilitate the implementation of the CFI, a number of measures were taken. First, a project group was formed that initiated and monitored the implementation efforts with direct communication lines with the organization’s leadership. Second, two forms of training were developed to prepare clinicians for the introduction of the CFI. The first training constituted an online training of approximately 60 minutes. The training focused on the rationale behind the CFI, its questions, and how it could be used and documented in the electronic patient file following clinical assessments. The online training did not address broader cultural competencies, beyond the CFI. The other course was designed as a training for supervisors, in which culturally competent members of the staff in different teams were trained to assist their own colleagues in the correct use of the CFI. This training focused on advanced background information on the CFI, frequent challenges when using the instrument, ways to incorporate the CFI in treatment planning, and finally skills in dealing with resistance among staff towards CFI use in routine practice. Both trainings were urgently recommended by the senior leadership, but were not mandatory to follow. The rationale was that mandatory training (a) would be too expensive to implement in a large organization, (b) could conflict with other scheduled training programs, and (c) might paradoxically decrease staff motivation to engage with the CFI. A third measure to facilitate the CFI implementation was a redesign of the standard intake format to accommodate the 16 CFI questions. As a result, patients’ responses to the CFI could be documented question by question in their electronic patient files, making it easier to find and use this information throughout the entire treatment process.

Despite these efforts, a preliminary evaluation of the CFI implementation revealed a number of concerning findings. The CFI appeared to be administered only in roughly 10% of all intake assessments. Moreover, an initial screening of these assessments revealed that in many cases the CFI responses were not documented completely or correctly.

These preliminary findings prompted a more thorough and stepwise investigation of potential barriers to CFI implementation. In the first step (study 1), we focused on the perception of the CFI by the staff, and organizational factors that were perceived to facilitate or hamper its correct application in the clinical practice. In the second step (study 2), we shifted away from organizational barriers and focused instead on patient and clinician characteristics that were associated with a more adequate use of the CFI during routine clinical assessments.

### Study 1

Following the initial implementation of the CFI, and some preliminary data indicating its limited use in routine clinical assessments (see above), the first study was conducted to investigate how the CFI was perceived by clinicians and key stakeholders, and which barriers they recognized for its application in daily practice.

#### Design

This study used a mixed-method methodology, with an explanatory sequential design (22), in which follow-up, qualitative interviews were conducted to better contextualize and understand results of a larger quantitative survey.

#### Participants

All 770 clinicians, working at an outpatient department of Parnassia Psychiatric Institute were approached to complete the survey. A total of 150 clinicians (19.5%) responded and were included in this study, among which 60 junior psychologists (40.0%), 54 senior psychologists (36.1%), 15 psychiatrists (10.0%), 10 psychiatric nurses (6.7%), and 11 other professionals (7.3%). In addition, semi-structured interviews were conducted with a number of clinicians and key stakeholders (i.e., administrators, experts and researchers) in order to get a more in-depth understanding of participants’ views on the CFI. A total of 13 clinicians and nine stakeholders were approached for the semi-structured interviews, of which eight clinicians (one psychiatrist, two senior psychologists and five junior psychologists) and eight stakeholders (board members, administrators and experts) agreed to participate. The participating clinicians were working at one of the main outpatient facilities with the greatest diversity in the number of departments and services provided. Respondents were approached based on their professional discipline to ensure all disciplines were represented in the interview sample.

#### Measures

##### Survey questionnaire

A survey questionnaire, specially developed for the purpose of this study, assessed clinicians’ perception of and attitude towards the CFI, and how they deployed it in everyday practice. The survey was administered online and consisted of 11 questions which could be answered on a 5-point Likert scale, ranging from “strongly disagree” to “strongly agree”. Examples of the survey items were: “Does the CFI have added value for your work?”, “Do you have enough time to complete the CFI during intake?”, and “Does the CFI help you to connect with the patient’s experience?”. Additionally, the survey contained three open ended questions inquiring about the value of the CFI and areas of improvement.

##### Semi-structured interviews

The interviews contained 10 items that inquired about the same topics as the survey, but also focused more specifically on the integration of the CFI in the standard intake assessment and the process of implementation [see **Appendix**]. The interviews were conducted, recorded and transcribed by the first author. An average interview took approximately 45-60 minutes to complete.

#### Procedure

The study was announced by the organization leadership, requesting the staff to complete a survey about their experience and perceptions of the CFI. Subsequently, an email invitation was sent to all clinicians working at an outpatient team to complete the online survey. A reminder was sent after two weeks, after which clinicians were given another two weeks to complete the survey. The survey was administered anonymously, and the respondents were not required to provide any personal information, except for their job title.

#### Analysis

Quantitative data were summarized by calculating the percentage of the respondents that endorsed key statements on the survey questionnaire. The interview data were analyzed using thematic analysis (23), a method that enables the systematic identification, organization, and interpretation of patterns within qualitative data. Initially, the data were repeatedly reviewed to ensure thorough familiarization with the content. Subsequently, the data were coded systematically. This involved assigning labels to segments of the text that appeared relevant or significant to the research questions. Both pre-determined themes, based on prior expectations, and emerging themes, which arose directly from the data, were used to guide the coding process. Once coding was completed, the data were categorized into coherent themes. This involved organizing the codes into broader themes that highlighted key patterns and insights. The themes were carefully refined to ensure they accurately represented the data and provided meaningful insights into the research questions.

#### Results

**Table 1** summarizes the main study findings. The majority of clinicians (66.9%) reported regular use of the CFI, as indicated by the online survey. The survey data, however, provided no information on the quality and fidelity of the CFI assessments. The remaining 33.1% did not use the CFI. The interview and the survey data revealed two main themes that will be further elaborated below: a) Information, training and barriers to implementation, and b) attitudes towards the CFI.

**Table 1:** Outcome of the online survey on the use and perception of the CFI (study 1).

Subcategories online survey	
*The use of the CFI*	%
Clinicians using the CFI	66.9
Clinicians not using the CFI	33.1
Background knowledge	
Fully informed about the CFI	26.4
Partially informed about the CFI	42.4
Not informed about the CFI	31.2
Training resources	
Familiar with online training	59.2
Followed the online training	41.3
Attitude	
Believe that the CFI has added value to the intake	6.9
Partial added value	45.2
No added value Only added value when working with ethnic minority patients	47.9 59.9

#### Information, training and barriers to implementation

##### Survey data

Only 26.4% of the clinicians who completed the online survey indicated that they were fully informed about the CFI during the implementation process, 42.4% indicated that they were partly informed, and 31.2% said not to have been informed at all. The clinicians were informed about the CFI mainly through their administrators, the online training and co-workers. Only around 40% of the respondents said to have completed the online training, while another 40% indicated not to have been aware that such a training existed. According to the survey data, reasons provided most for not completing the online training were insufficient time and not knowing that the training existed.

##### Interview data

Clinicians. Many clinicians reported administering the CFI as a structured interview, resulting in a diminished sense of rapport with their patients during intake assessments, instead of fostering it. Reflecting on the possible reasons, many pointed to the limited information that they had received about the CFI, and its underlying idea and rationale, as illustrated by the following quote:

“I did not receive any information about the CFI from my manager. I read about the online training [only] on the intranet.”

Another reason the clinicians provided during the interviews was that both clinicians and patients were overwhelmed by the large number of questions to be covered during intake assessment. This allowed insufficient time and flexibility to use the CFI as a tool to immerse in patients’ context and perspective. One respondent described this problem as follows:

“Because of the CFI I am so focused on all the questions that need to be asked that I lose contact with my patient. The CFI feels like a checklist to me.”

A final reason that the clinicians provided was that the intake assessment, including the CFI, was often conducted by junior psychologists who were predominantly trained in symptomatic assessment and treatment. An instrument, such as the CFI, that focused on the perspectives and sociocultural context of the patients did not fit with their prior training and competencies.

Key stakeholders. The key stakeholders identified similar barriers to implementation. According to them, a major reason for the flawed implementation of the CFI was the absence of a shared understanding of the CFI as an instrument that can improve the clinical assessment and rapport across the organization. Consequently, actors within different layers of the organization were not equally committed to its implementation. As a matter of fact, several administrators did not support the implementation of the CFI within the organization (see the section on attitudes towards the CFI for more details). This lack of a shared commitment led, in turn, to a tentative implementation policy. The respondents criticized, for example, the policy in which the CFI trainings (online training as well as the training of supervisors) though urgently recommended, was not regarded as mandatory. Also, the timing of the implementation was viewed as unfortunate, given a number of other significant changes in the organization. Several administrators, for example, concluded during the interviews that they did not pay enough attention to the implementation of the CFI in their department, due to other ongoing projects, i.e., the introduction and implementation of DSM-5. Finally, the key stakeholders concluded that the large scale of the organization made it difficult to implement a change that suits all departments.

#### Attitudes towards the CFI

##### Survey data

Despite the fact that 66.9% of the respondents of the online survey indicated that they used the CFI, the general attitude towards the CFI was not positive. In fact, only 6.9% believed that the CFI had an added value to the intake assessment. Almost half of all respondents (47.9%) saw little or no added value in the CFI. Finally, the majority of the clinicians (59.9%) believed that the CFI had only added value, when working with ethnic minority patients.

##### Interview data

Clinicians. Clinicians held mixed views on the relevance of the CFI. A number of clinicians believed that patients who seek mental health services for certain psychiatric disorders, such as attention deficit and hyperactivity disorder (ADHD), anxiety disorders and depression, generally benefit more from a symptom-oriented approach rather than a contextual one. Hence, the CFI was viewed as obsolete for this population. In contrast, patients with personality disorders could benefit more from a context-oriented treatment, which justified the use of the CFI among this group. For instance, one clinician noted:

“I don’t use the CFI often in intakes. Patients come to me for an ADHD treatment. It is not clear to me what to do with the information from the CFI during the treatment.”

Other clinicians, however, saw the importance of a contextual approach in all treatments, arguing that all patients live in their own unique context, and that taking this context into account is essential for the right diagnosis and treatment planning, as indicated in the following quote:

“I think it is very important to look at the context of the patient. I think that every relationship the patient has, is an intercultural relationship and that it is therefore necessary to look at the context or culture with all patients.”

Key stakeholders. Administrators who participated in the interviews displayed the same mixed attitude towards the CFI. Some believed that de CFI should be universally used for all patients, while others indicated that only specific patient groups could benefit from the CFI, such as immigrants, and those with complex psychiatric problems, i.e., personality disorders and severe posttraumatic stress disorder:

“The need to use the CFI may be less for a three-month manualized treatment for depression than for a treatment for a personality disorder. I therefore think the clinicians themselves should see how they want to use the CFI.”

In contrast, some other stakeholders believed that a focus on culture and context remain a fundamental part of every assessment. For example, one administrator stated:

“I think the context of the patient is very important. It’s good to know someone’s theory of illness. If you do not have a clear understanding of this, you are at risk of offering a treatment that does not meet the patient’s need for help and then the therapy will not work.”

These diverse views on the relevance of the CFI seemed, at least partially, to stem from different perspectives on the role of culture and context in clinical assessment and treatment planning. Some administrators argued that the main focus of assessment is to establish a psychiatric diagnosis, based on the presenting symptoms, which would inform a specific treatment. Others indicated that establishing a diagnosis inevitably entailed focusing on patients’ context and life circumstances. Only one interview participant mentioned explicitly that the CFI could help create a better rapport between patients and clinicians. Finally, concerning the CFI implementation, some administrators believed that it was mainly up to the clinician to decide whether the CFI should be used during an assessment or not. Thus, adopting a top-down organizational policy that enforced the use of the CFI was considered counterproductive.

#### Conclusions

In sum, the results pointed to the complexity of implementing the CFI within large scale mental health services. Barriers to implementation included clinicians’ lack of familiarity with the instrument and the rationale for its application as part of routine clinical assessment. Additionally, the study identified organizational barriers, including a lack of shared commitment to the implementation of the CFI and competing priorities within the institute.

Attitudes towards the CFI were diverse among clinicians and administrators and seemed partly inconsistent with the APA recommendations (14). While some acknowledged its potential benefits for certain patient populations, such as ethnic minorities and those with complex psychiatric conditions, the majority questioned its relevance, particularly in the context of symptomatic assessment and treatment.

Finally, and contrary to previous expectations (21), the results suggest that the mere introduction of the CFI may not suffice to promote person-centered assessment within mainstream services. The CFI runs the risk of becoming reduced to a mere checklist or a structured interview in a medical context that is not sufficiently open to a person- and context-oriented approach. This raises the question whether *clinicians’* pre-existing competencies may be crucial in determining how well they use the CFI. Among these competencies, clinicians’ *cultural* competencies (CCC) may be of great relevance to the implementation of the CFI. Thus, study 2 investigated a number of patient and clinician characteristics, associated with the adequate use of the CFI, including CCC.

### Study 2

The previous study pointed to a number of major challenges in the implementation of the CFI within a medical culture that is not fully receptive to a contextual and person-centered approach. Adopting the CFI in such a clinical context may pose significant threats to its fidelity. That is, the CFI may end up not being administered as intended, eventually leading to an inadequate cultural assessment. To gain further insight into CFI fidelity in clinical practice, the current study utilized a historical cohort data to examine which patient and clinician variables were associated with greater CFI fidelity during initial assessments. Of particular interest was the presumed link between CCC and CFI fidelity, meaning that more culturally competent clinicians would likely generate more adequate cultural assessments, using the CFI.

#### Design

This quantitative study used a cross-sectional design, with CFI-fidelity as the presumed dependent variable and CCC as the presumed independent variable.

#### Participants

The sample consisted of (a) consenting clinicians at an outpatient facility of Parnassia Psychiatric Institute who had completed intake assessments between September 2019 and April 2020 and (b) patients who were examined for an intake by one of these clinicians during that time frame and who had previously provided consent for the use of their data for research purposes. Clinicians were further eligible for inclusion if they conducted at least one intake assessment a week and were working for at least one year at the outpatient facility.

#### Measures

The following instruments were used in this study.

##### Cultural and linguistic competency self-assessment checklist for personnel providing primary health care services

CCC was measured with a translated version in Dutch of the cultural and linguistic competence self-assessment checklist Georgetown (24). The self-assessment checklist contains 37 items divided over three domains: (a) the physical environment (related to display of materials in the consultation room), (b) communication style and (c) values & attitudes. The checklist uses a three-point Likert scale, measuring the frequency with which clinicians applied certain behaviors or attitudes in contact with patients with diverse cultural backgrounds. The total score expresses the degree to which the clinicians have competences that enable effective work in cross-cultural situations. Before running the analysis, the questions pertaining to the physical environment were removed from the data analysis, because this information was the same for all clinicians since they share the same environment to treat the patients.

##### Cultural Formulation Interview Rating Scale

CFI-fidelity was assessed, using a rating scale that was specifically developed for the current study. The CRS comprises five different modules, corresponding to the different domains of the CFI (1): cultural definition of the problem and explanatory models of illness (2), stressors and support (3), cultural identity (4), cultural factors affecting coping and past help seeking, and finally (5) cultural factors affecting current help seeking. The electronic patient file provided separate fields for each of the 16 CFI questions. For each module the fidelity of the CFI information, as documented in the electronic patient file, was independently assessed by two reviewers (KA and SG), using a three-point rating scale that was based on the following operationalization: The content of the module in the electronic patient record was assigned a score of 2, if full information was available on all CFI questions within that module. A score of 1 was assigned to descriptions that did not cover the entire module. In other words, the description contained some relevant elements, but not all CFI questions were addressed in the description. Finally, a score of 0 was assigned to descriptions that lacked clarity, did not address relevant topics within a module, or contained no cultural or contextual information at all. The total CRS score is a measure of the degree of fidelity of the CFI reports.

The scoring criteria, outlined above, were developed in close cooperation with two experts on cultural formulation. Subsequently, a pilot assessment was conducted, using ten CFI reports, which were scored by two independent reviewers (AK and SG). This was followed by comparing the results and evaluating the degree of agreement or disagreement on the scores between the reviewers. Next, the reviewers analyzed and discussed the origins of the differences, and the criteria of the rating scale were adapted where necessary. This procedure was repeated a total of four times before arriving at the final version of the instrument. The interrater agreement of the final version was 95%.

##### Demographic questionnaires

###### Clinicians

A five-item self-reported questionnaire was used to collect clinicians’ demographic information, such as gender, age, ethnicity, professional discipline and years of experience as a mental health professional.

###### Patients

Patient demographic information was extracted from their electronic records and contained data on gender, age, ethnicity, relationship status, number of children, level of education, employment status, Global Assessment of Functioning (GAF), diagnoses according to DSM-5, and the presence or absence of Comorbidity.

#### Procedure

A total of 21 clinicians were invited to participate in the study and to fill out the questionnaires on demographics and CCC. Ten clinicians (47.6%) returned the questionnaires, and thus were included in the study. These clinicians had conducted a total of 145 intake assessments during the study period. Thirty-three assessments were discarded: 27 electronic records could no longer be accessed, and six records had incomplete demographic information. The remaining 112 patients (records) were included in the study.

#### Analysis

First, a bivariate (Pearson) correlation analysis was performed with CFI-fidelity, the CCC and all demographic variables. Demographic variables with significant correlations and no multicollinearity effect were selected for inclusion in the main analyses of CFI-fidelity and CCC. Next, a multiple regression analysis (method enter) was conducted with CFI-fidelity as outcome and significantly correlated demographic variables (step 1), and CCC (step 2) as predictors. The data analysis was performed with SPSS, version 26 (25).

#### Results

##### Sample characteristics

The sample characteristics are presented in **Table 2**. The majority of the patients were female with a native Dutch background and had at least completed secondary education. Most participating clinicians were female with a native Dutch ethnicity, had a background in clinical psychology and had between three to five years of experience in mental health care. There was no information available on the number of clinicians who had followed the online CFI training in the past.

**Table 2:** Sample characteristics of study 2.

Patients	N	%
Gender		
Male	50	45
Female	62	55
Ethnicity		
Dutch	90	80
Non-Dutch	22	20
Education		
Low	6	5
High school	35	32
Technical	47	42
Higher	13	12
No information	10	9
Relationship status		
In a relationship	66	59
Single	46	41
Number of children		
None	68	61
1 or more	44	39
Employment		
Yes	73	65
No	39	35
DSM-5 classification		
Personality Disorder	16	14
Anxiety Disorder	7	6
PTSD	13	12
ADHD	38	34
Depressive Disorder	19	17
Feeding and eating Disorder	2	2
Substance related addictive Disorder	2	2
Gender Dysphoria & sexual Dysfunction	15	13
Comorbidity		
No	6	38
1 Or more	76	62
Age	(M) 34.12	(SD) 9.85
Clinicians	N	%
Gender		
Male	1	10
Female	9	90
Ethnicity		
Native Dutch	8	80
Non-Native	2	20
Professional Background		
Psychologist	9	90
Nurse practitioner	1	10
Working experience (in years)		
2-5	7	70
5-10	2	20
45	1	10
Age	(M) 31	(SD) 10.58

##### Association between CFI fidelity, CCC and demographic variables

The correlation analysis showed a positive association between CCC and CFI-fidelity (*r* =.35) (see **Table 3**), suggesting that more culturally competent clinicians generated more adequate cultural assessments reports, using the CFI. Furthermore, clinician’s age, patient’s age, patient’s relationship and employment status had a significant correlation with CFI fidelity. Specifically, older clinicians produced more adequate cultural assessment reports. Further, CFI fidelity was higher for older patients and those who were in a relationship or unemployed at the time the study.

**Table 3:** Correlations between CFI fidelity and other variables (study 2).

	1	2	3	4	5	6	7	8	9	10	11	12	13
1. CFI-fidelity	–												
2. Clinician's Cultural Competences (CCC)	.35**												
3. Clinician's gender	-0.03	-.19*											
4. Clinician's age	.26**	0.11	-.53**										
5. Clinician's ethnicity	-.4**	-.53**	-0.16	-.27**									
6. Clinician's work experience in Mental Health	0.15	-.27**	-.58**	.75**	0.06								
7. Patient's gender	0.03	0.13	.19*	-0.16	-0.1	-.19*							
8. Patient's age	-.18**	-0.07	0.13	-0.07	0.06	-0.03	-0.16						
9. Patient's ethnicity	0.11	0.08	0.02	-0.14	-0.03	-0.04	0.12	0.04					
10. Patient's relationship status	**.21**	0.05	0	-0.03	-0.11	-0.08	0.02	-.42**	-0.16				
11. Patient's number of children	-0.15	-0.14	0.13	-0.08	0.08	-0.01	-0.01	.65**	0.15	-.52**			
12. Patient's education level	-0.17	-0.04	0.14	-0.12	0.06	-0.01	0.02	0.09	-0.12	0	-0.11		
13. Patient's employment status	-.19*	0.01	-0.07	0	0.04	0	-0.12	0.12	-0.02	-0.15	-0.04	.18*	
14. GAF-Global Assessment of Functioning	-0.08	-0.09	-0.15	-0.07	0.13	-0.06	0.08	-0.16	-.19*	0.06	-0.17	0.01	0.13

*p < .05; **p < .01. Bold indicates significant correlation.

##### Relationship between CCC and CFI-fidelity

In the first step of the multiple regression analysis, entering clinician’s age, patient’s age, patient’s relationship status, and patient’s employment status, resulted in a model which explained 9% of the variance in CFI fidelity. Adding CCC explained an additional 4% of variance in CFI fidelity. The final model was significant, *F* (3, 352), *p* <.01.

As hypothesized, CCC showed a positive association with CFI-fidelity. Among demographic variables, the patient’s employment status was the only factor that was still significantly associated with CFI fidelity. This indicated that cultural assessments were more adequate for patients who were unemployed (see **Table 4**).

**Table 4:** Regression analysis of factors, predicting CFI fidelity (study 2).

		Unstandardized Coefficients		Standardized Coefficients
		B	SE	*Beta*
1	(Constant)	.60	.19	
	Clinician’s age	.00	.00	.09
	Patient’s age	.00	.00	-.10
	Patient’s relationship status	.09	.07	.12
	Patient’s employment status	-.15	.07	-.19
2	(Constant)	.14	.28	
	Clinician’s age	.00	.00	.04
	Patient’s age	.00	.00	.09
	Patient’s relationship status	.11	.07	.14
	Patient’s Employment status	-.15	.07	-.20*
	Clinician Cultural Competences	.26	.12	.20*

*p < 0.05.

#### Conclusion

Study 2 was conducted to shed light on factors that may potentially impact CFI fidelity. Analyzing a host of patient and clinician variables, only two factors significantly predict CFI fidelity in this sample. First, CFI fidelity was higher for patients who were unemployed at the time of the study. This is explained by the fact that unemployment is generally considered a major risk factor for mental distress, and could be correlated with other psychosocial stressors, such as family problems (26). Therefore, it is plausible that patients’ unemployment status may have prompted clinicians to investigate psychosocial stressors more thoroughly, eventually producing a more adequate cultural and contextual assessment. The second factor associated with CFI fidelity was CCC. As predicted, more culturally competent clinicians produced more adequate cultural assessments, using the CFI. This finding may have important implications for CFI implementation efforts within mainstream services. We shall elaborate more on these implications in general discussion.

## General discussion

This paper reported on two studies that were carried out in the context of large-scale implementation of the CFI in mainstream psychiatric services in the Netherlands. Following preliminary findings, suggesting the CFI was insufficiently used during routine intake assessments or not used as intended, the two studies focused on how the CFI was perceived by staff and stakeholders, and which factors were associated with higher CFI fidelity. In summary, the findings highlighted the challenges involved in integrating the CFI into large-scale mental health services. Obstacles to implementation encompassed clinicians’ limited familiarity with the interview and its purpose in routine clinical assessments. Moreover, organizational hurdles, such as a lack of unified commitment to CFI implementation and competing institutional priorities, were identified. Attitudes towards the CFI varied among clinicians and administrators, deviating somewhat from APA recommendations, emphasizing its application for all patients and in all settings (14). While some recognized its potential advantages for specific patient groups, such as ethnic minorities and individuals with complex psychiatric conditions, many questioned its relevance, particularly in the context of symptom-focused assessment and treatment. Finally, we found evidence of a direct connection between CCC and CFI fidelity, suggesting that more culturally competent clinicians were better able to use the CFI as intended.

Overall, the results underscore significant challenges in implementing the CFI on a large scale within routine clinical practice. Compared to the results of the DSM-5 field trials (19), our two studies revealed lower CFI fidelity during intake assessments and more negative perceptions of the interview among the treatment staff. Although the CFI has been designed as a clinical tool to be easily integrated in a standard intake assessment, our data suggest that its implementation may be hampered by a host of (interrelated) factors. Firstly, since the integration of the CFI in routine assessment entails a fundamental shift towards a person-centered and contextual approach (21), a lack of sustainable commitment across all layers of the organization may be detrimental to its implementation process. Ideally, this commitment stems from a shared appreciation of the cultural formulation approach as a core element of a standard assessment. Secondly, rapid implementation efforts, coinciding with other organizational changes diminish the commitment to adopting the CFI as intended. This concurrent focus on multiple initiatives dilutes the attention and resources available for the successful integration of the CFI. Thirdly, the absence of mandatory training opportunities restricts staff members from fully grasping the utility and feasibility of the CFI in their practice. This can, in turn, contribute to the perception of the CFI as a burden rather than a valuable resource.

Finally, our findings suggest an association between staff members’ cultural competencies and their proficiency in using and documenting the CFI. This has an important ramification in light of current assumptions regarding the CFI. Specifically, our finding challenges the idea that the CFI, as a primary catalyst, can help implement and solidify culturally competent practices within mainstream services. According to our data, integrating the CFI into routine practice appeared to advance cultural assessments more effectively among clinicians who already possessed stronger cultural competencies compared to others. Thus, focusing on the CFI as the main “agent of change” (21) may prove futile, as clinicians may require a broader range of cultural competencies to effectively utilize the CFI as intended.

Several limitations are worth noting in both studies. Firstly, concerns arise regarding the generalizability of the findings. The samples in both studies comprised only a small percentage of the entire organizational staff, and all participants were exclusively from outpatient facilities. Consequently, there is considerable uncertainty about whether the results can be extended to other clinical or outreach services. Furthermore, in Study 1, the relatively low survey response rate (19.5%) might have introduced a response bias, as individuals with more extreme attitudes toward the CFI were more likely to participate. The low response rate could be attributed to a high workload among clinicians and the simultaneous introduction of the DSM-5, making the evaluation of the CFI a lower priority for clinicians. For the interviews, 22 clinicians and stakeholders were approached, with 16 participating, which is a relatively small subsample. Similarly, in Study 2, although a substantial number of intake assessments were analyzed (N = 112), they were conducted by a limited number of clinicians (N = 10). Finally, CFI fidelity was assessed solely based on the documentation of relevant CFI information in electronic patient files. There was no information on how CFI topics were addressed during intake assessments or their impact on final diagnosis and treatment planning. Future implementation studies should address these limitations by examining the specific impact of the CFI on clinical processes, preferably using larger and more diverse samples.

Despite these limitations, the two studies comprise the first attempt to date to evaluate a large-scale implementation of the CFI, and one of the few attempts so far to document its use among ethnic majority patient populations in the Global North. Based on our findings, the following implications seem warranted. First, the implementation of the CFI in routine clinical assessment requires a fundamental rethinking of the overall assessment process to incorporate cultural and contextual information. This is a slow and delicate process, not amenable to rapid implementation on a grand scale. For larger organizations, it is advisable to implement the CFI on a small scale, allowing a limited number of clinicians to gain positive experience with the cultural formulation, and to gradually expand and refine implementation efforts, based on initial experiences. Lastly and more importantly, it is imperative to prioritize the overarching goals of the implementation process over the specific tools employed. The CFI is a mere instrument to establish a cultural formulation of diagnosis, not an objective in itself. Overemphasizing the CFI may lead to a counterproductive instrumentalization of the cultural formulation. This may, in turn, result in using the CFI as a mere checklist to be completed during assessment with minimal impact on diagnosis and treatment planning. In line with our findings, one important way to mitigate this risk is to train clinicians in broader cultural competencies rather than solely focusing on the CFI.

## References

[1] Kirmayer LJ Ryder AG. Culture and psychopathology. Curr Opin Psychol. (2016) 8:143–8. doi: 10.1016/j.copsyc.2015.10.020 29506790

[2] Kleinman A Benson P. Anthropology in the clinic: the problem of cultural competency and how to fix it. PLoS Med. (2006) 3:e294. doi: 10.1371/journal.pmed.0030294 17076546 PMC1621088

[3] Kleinman A. Patients and Healers in the Context of Culture: An Exploration of the Borderland Between Anthropology, Medicine, and Psychiatry. Berkeley: University of California Press (1980).

[4] Comas-Díaz L. Cultural variation in the therapeutic relationship. In: Goodheart CD Kazdin AE & Sternberg RJ, editors. Evidence-based psychotherapy: Where practice and research meet. American Psychological Association, Washington, DC (2006). p. 81–105.

[5] Soto A Smith TB Griner D Domenech Rodríguez M Bernal G. Cultural adaptations and therapist multicultural competence: Two meta-analytic reviews. J Clin Psychol. (2018) 74:1907–23. doi: 10.1002/jclp.22679 30091201

[6] Griner D Smith TB. Culturally adapted mental health intervention: A meta-analytic review. Psychotherapy. (2006) 43:531–48. doi: 10.1037/0033-3204.43.4.531 22122142

[7] Benish SG Quintana S Wampold BE. Culturally adapted psychotherapy and the legitimacy of myth: a direct-comparison meta-analysis. J Couns Psychol. (2011) 58:279–89. doi: 10.1037/a0023626 21604860

[8] Ahmad G McManus S Cooper C Hatch SL Das-Munshi J. Prevalence of common mental disorders and treatment receipt for people from ethnic minority backgrounds in England: repeated cross-sectional surveys of the general population in 2007 and 2014. Br J Psychiatry. (2022) 221:520–7. doi: 10.1192/bjp.2021.179 PMC7616892 35049474

[9] APA. Diagnostic and Statistical Manual of Mental Disorders, 4th Edition (DSM-IV). Washington, DC: APA (1994).

[10] Mezzich JE. Cultural formulation and comprehensive diagnosis. Clin Res perspectives Psychiatr Clin North Am. (1995) 18:649–57. doi: 10.1016/S0193-953X(18)30046-7 8545273

[11] Rohlof H Knipscheer JW Kleber RJ. Use of the cultural formulation with refugees. Transcult Psychiatry. (2009) 46:487–505. doi: 10.1177/1363461509344306 19837783

[12] Bäärnhielm S Scarpinati Rosso M. The cultural formulation: A model to combine nosology and patients’ Life context in psychiatric diagnostic practice. Transcult Psychiatry. (2009) 46:406–28. doi: 10.1177/1363461509342946 19837779

[13] Aggarwal NK Jarvis GE Omez-Carrillo AG Kirmayer LJ Lewis-Fernández R. The Cultural Formulation Interview since DSM-5: Prospects for training, research, and clinical practice. Transcultural Psychiatry (2020) 57(4):496–514. doi: 10.1177/1363461520940481 32838655

[14] American Psychiatric Association. Diagnostic and Statistical Manual of Mental Disorders (DSM-5). Washington, DC: APA (2013).

[15] Aggarwal NK Cedeno K Lewis-Fernandez R. Patient and clinician communication practices during the DSM-5 cultural formulation interview field trial. Anthropol Med. (2020) 27:192–211. doi: 10.1080/13648470.2019.1641014 31550913 PMC7093248

[16] Aggarwal NK DeSilva R Nicasio AV Boiler M Lewis-Fernández R. Does the Cultural Formulation Interview (CFI) for the Fifth Revision of the Diagnostic and Statistical Manual of Mental Disorders (DSM-5) affect medical communication? A qualitative exploratory study from the New York site. Ethn Health. (2015) 20:1–28. doi: 10.1080/13557858.2013.857762 25372242 PMC4221811

[17] Ramírez Stege AM Yarris KE. Culture in la clínica: Evaluating the utility of the Cultural Formulation Interview (CFI) in a Mexican outpatient setting. Transcult Psychiatry. (2017) 54:466–87. doi: 10.1177/1363461517716051 28691591

[18] Brand AM Groen SP Destoop N Jongsma HE Ghane S Sabbe BG. The effect of the cultural formulation interview on therapeutic working alliance: a study protocol. Front Psychiatry. (2024) 15:1322356. doi: 10.3389/fpsyt.2024.1322356 38501082 PMC10945007

[19] Lewis-Fernández R Aggarwal NK Lam PC Galfalvy H Weiss MG Kirmayer LJ. Feasibility, acceptability and clinical utility of the Cultural Formulation Interview: Mixed-methods results from the DSM-5 international field trial. Br J Psychiatry. (2017) 210(4):290–7. doi: 10.1192/bjp.bp.116.193862 28104738

[20] Aggarwal NK Glass A Tirado A Boiler M Nicasio A Alegría M. The development of the DSM-5 cultural formulation interview-fidelity instrument (CFI-FI): A pilot study. J Health Care Poor Underserved. (2014) 25:1397–417. doi: 10.1353/hpu.2014.0132 PMC4306341 25130248

[21] Lewis-Fernandez R Aggarwal NK Kirmayer LJ. Introduction. In: Lewis-Fernandez R Aggarwal NK Hinton L Hinton DE Kirmayer LJ, editors. DSM-5 Handbook on the Cultural Formulation Interview. American Psychiatric Publishing, Washington, DC (2016). p. xxvii–xiv.

[22] Creswell JW Zhang W. The application of mixed methods designs to trauma research. J Traumatic Stress: Off Publ Int Soc traumatic Stress Stud. (2009) 22.6:612–21. doi: 10.1002/jts.20479 19960518

[23] Braun V Clarke V. Using thematic analysis in psychology. Qual Res Psychol. (2006) 3:77–101. doi: 10.1191/1478088706qp063oa

[24] Goode T. Promoting Cultural and Linguistic Competency Self-Assessment Checklist. Berkeley: National Center for Cultural Competence, Georgetown University Center for Child & Human Development (2009).

[25] George D Mallery P. IBM SPSS statistics 26 step by step: A simple guide and reference. Milton Park: Routledge (2019).

[26] Erdem Ö Van Lenthe FJ Burdorf A. Income inequality and psychological distress at neighbourhood and municipality level: An analysis in the Netherlands. Health Place. (2019) 56:1–8. doi: 10.1016/j.healthplace.2018.12.011 30660742

