# Supplementary material for: Symptom vs context: lessons learned from a large-scale implementation of the Cultural Formulation Interview
Source: Front Psychiatry. 2024 Sep 4;15:1410865. doi: 10.3389/fpsyt.2024.1410865 (PMC11408998; doi:10.3389/fpsyt.2024.1410865)
Supplement: Supplementary file 1 [file DataSheet1.docx]

| Appendix 1. Qualitative Interview questions (study 1) |
| --- |
|  |
| 1. Why do you think it was important for Parnassia Psychiatric Institute to implement the CFI in the intake assessment? |
|  |
| 1. What are your thoughts on the implementation process of the CFI so far? |
|  |
| 1. At this moment, both symptoms and context of the patient are examined in the intake assessment. What is your view on this? What is the purpose of the intake in your opinion and how can we achieve this? |
|  |
| 1. Do you think that the approach in the intake should be the same for all patient groups (e.g. Personality disorders, depression or ADHD)? |
|  |
| 1. Is there also added value for the CFI in departments that mainly provide manualized treatments? |
|  |
| 1. What role do you think the CFI will have in the future within Parnassia Psychiatric Institute? |
|  |
| 1. There are different opinions about the added value of the CFI in the intake assessment. What is your perspective on this? |
|  |
| 1. The CFI is now implemented in the intake assessment. How do you see the role of the CFI during treatments? |
|  |
| 1. What do you think are three strong points of the CFI? |
|  |
| 1. What do you think are three areas for improvement of the CFI? |
|  |
| For clinicians only: |
| 1. How do you use the CFI yourself and what is the effect of the CFI in your contact with the patient? |
